# Transcriptomic Analysis of Bovine Oocytes at GV and MII Stages and Dynamic Changes in Key Gene Expression Patterns

**DOI:** 10.3390/biology15080662

**Published:** 2026-04-21

**Authors:** Xueyan Wang, Fei Huang, Xiaopeng Li, Kai Hu, Hong Chen, Peng Niu, Huimin Qu, Di Fang, Chunmei Han, Qinghua Gao

**Affiliations:** 1 College of Animal Science and Technology, Tarim University, Alar 843300, China; wangxueyan1016@163.com (X.W.);; 2 College of Life Science and Technology, Tarim University, Alar 843300, China; 3 Key Laboratory of Tarim Animal Husbandry Science and Technology, Xinjiang Production & Construction Corps, Alar 843300, China

**Keywords:** oocyte maturation, single-cell RNA-seq, RT-qPCR, in vitro maturation

## Abstract

The maturation of oocytes is a critical step in mammalian reproduction, involving dynamic regulation of gene expression. Therefore, investigating how gene expression varies during different stages of oocyte maturation is highly important. This study employed single-cell RNA sequencing (scRNA-seq) to analyze bovine oocytes at the germinal vesicle (GV) and metaphase II (MII) stages. The results identified 1787 differentially expressed genes (DEGs) between the two stages, with 1556 genes upregulated and 231 downregulated in the GV stage. Further investigation using Gene Ontology (GO) and Kyoto Encyclopedia of Genes and Genomes (KEGG) enrichment analyses indicated that the upregulated genes are mainly involved in mitochondrial functions and energy metabolism, whereas the downregulated genes are primarily associated with signaling pathways. Validation through RT-qPCR confirmed that *COA4*, *TKT* and *GPX4* were significantly higher in GV-stage oocytes, while *ISG15*, *MAP1LC3C* and *ZEB2* were notably downregulated. This study highlights significant gene expression differences between GV and MII bovine oocytes, underscoring the vital roles of genes related to energy metabolism and signaling during oocyte maturation. The expression patterns of these genes provide important molecular markers for further elucidating the mechanisms underlying oocyte maturation.

## Simple Summary

The maturation of oocytes is essential for successful reproduction in mammals; however, the underlying molecular mechanisms remain incompletely understood. This research utilized Smart-seq2 sequencing to examine gene expression variations in bovine oocytes during two key developmental stages: the germinal vesicle (GV) and metaphase II (MII). We identified numerous genes that exhibited significant differences in expression between these stages. Many of these genes are associated with mitochondrial function, energy metabolism and signaling pathways, all of which are crucial for the development of oocytes. Furthermore, we confirmed the expression patterns of several key genes, which varied throughout the maturation process. These findings deepen our comprehension of the molecular basis of oocyte maturation and may inform strategies to improve oocyte quality and reproductive success in livestock.

## 1. Introduction

Oocyte maturation is a central event in mammalian reproduction, and typically only one or two oocytes successfully complete this process [1]. This process initiates at the germinal vesicle (GV) stage, where oocytes are arrested in prophase I of meiosis. Once the germinal vesicle breaks down (GVBD), meiosis resumes, leading oocytes into the nuclear maturation stage characterized by chromosome condensation, nuclear envelope breakdown and spindle apparatus formation [1,2]. Subsequently, oocytes complete the first meiotic division, release the first polar body, and pause at metaphase II (MII) [2]. During this process, nuclear and cytoplasmic maturation proceed in a coordinated manner and are regulated by multiple layers of factors, including endocrine hormonal signaling [3,4], intracellular metabolic status [5,6], intercellular communication [7,8] and gene expression regulation [9,10,11].

During oocyte maturation, gene expression undergoes pronounced dynamic changes. The selective degradation of maternal mRNAs, temporal regulation of specific developmental genes and the involvement of non-coding RNAs collectively form a complex regulatory network governing this process [12,13,14,15]. Cross-species studies further indicate that gene expression during oocyte maturation exhibits both conserved and species-specific features [16,17], ensuring the accurate transmission of maternal genetic information to the next generation post-fertilization. These findings underscore the importance of understanding the transcriptomic landscape of bovine oocytes.

As meiosis resumes, transcription in oocytes ceases, leading to the selective breakdown of maternal mRNAs and a transition in gene expression regulation from nuclear transcription to cytoplasmic translation [1]. Simultaneously, oocyte maturation encompasses several energy-intensive biological activities, such as chromosome segregation, spindle formation, and organelle restructuring, with energy primarily derived from glycolysis, the tricarboxylic acid cycle and oxidative phosphorylation (OXPHOS) [8,18,19]. In this context, OXPHOS is identified as the principal source of ATP production, providing the necessary energy for oocyte maturation [20]. However, it is essential to carefully regulate the levels of reactive oxygen species (ROS) generated during these metabolic processes [21]. On the one hand, an increase in luteinizing hormone (LH) triggers a significant rise in ROS, which act as signaling molecules to modulate oocyte maturation, ovarian steroid production, corpus luteum activity, and luteolysis; on the other hand, an excessive accumulation of ROS can lead to meiotic abnormalities, alterations in spindle microtubules, and chromosome misalignment [22]. Therefore, precise regulation of ROS homeostasis is vital for maintaining oocyte viability and ensuring successful fertilization.

The rapid advancement of high-throughput sequencing methods has enabled single-cell RNA sequencing (scRNA-seq) to analyze dynamic changes in transcriptomes within small groups of cells or even at the level of individual cells, leading to its widespread application in studies of oocyte development [23]. Previous research employing scRNA-seq has revealed crucial information about the transcriptomic profiles of oocytes at different meiotic stages across various mammals, including humans [24], pigs [25], sheep [26], and donkeys [27]. Additionally, recent studies focusing on bovine oocytes have primarily addressed conditions such as vitrification [28,29], hormonal supplementation [30] and heat stress [31]. Therefore, this study employed scRNA-seq to analyze the transcriptomic profiles of bovine oocytes at the GV and MII stages, aiming to characterize stage-specific gene expression patterns and identify candidate genes and regulatory mechanisms underlying oocyte maturation. Finally, the expression of selected candidate genes was validated by real-time quantitative PCR (RT-qPCR), providing a theoretical basis for improving bovine oocyte developmental competence.

## 2. Materials and Methods

### 2.1. Bovine Ovary Collection

Ovaries were obtained from adult female Simmental cattle at Xiyu Livestock Co., Ltd. in Aksu, Xinjiang, China, during routine slaughtering. The collected ovaries were rinsed with 75% ethanol and placed in a thermos filled with physiological saline maintained at 37 °C, which contained 100 IU/mL of penicillin and 100 IU/mL of streptomycin. Upon arrival at the laboratory within 3 h, excess connective tissue surrounding the ovaries was removed using sterilized surgical scissors. The ovaries were then washed three times with physiological saline at 37 °C and maintained in physiological saline at 37 °C until further processing.

### 2.2. Oocyte Collection and In Vitro Maturation

Follicular fluid from follicles with a diameter of 3–8 mm was aspirated using a disposable syringe and collected into a sterile 60 mm culture dish. Under a stereomicroscope (Nikon, Tokyo, Japan), cumulus–oocyte complexes (COCs) surrounded by at least three layers of cumulus cells were selected using a mouth pipette and transferred to pre-equilibrated in vitro maturation medium (71001, HARIOMED, Guangzhou, China) for three washes. Subsequently, groups of 15–20 oocytes were transferred to 80 μL droplets of in vitro maturation medium in a culture dish and covered with mineral oil to prevent evaporation. The oocytes were then cultured for 24 h in a CO_2_ incubator (Sanyo, Tokyo, Japan) at 38.5 °C with 5% CO_2_ and saturated humidity.

To obtain GV-stage oocytes, cumulus cells were removed from the collected COCs using 0.1% hyaluronidase. For MII-stage oocytes, cumulus cells were removed after 24 h of in vitro maturation using the same procedure and extrusion of the first polar body was used as the indicator of successful oocyte maturation.

### 2.3. RNA Extraction, cDNA Library Preparation, and Sequencing

GV- and MII-stage oocytes were directly collected into cell lysis buffer provided by BGI (Shenzhen, China). Full-length cDNA synthesis was performed using the SMART-Seq2 protocol, which combines oligo (dT) primers and a template-switching mechanism to efficiently capture polyadenylated RNA transcripts from single cells. After reverse transcription, the cDNA was pre-amplified by PCR to obtain sufficient material for subsequent library construction.

Library construction was performed using a transposase-mediated tagmentation method, which simultaneously fragments the cDNA and ligates sequencing adapters and indices. The constructed libraries underwent quality control before sequencing, with a focus on insert size distribution and concentration. After amplification, the cDNA products were denatured into single strands, followed by a circularization reaction to generate single-stranded circular DNA molecules. These circular DNA molecules were subjected to rolling-circle replication to form DNA nanoballs (DNBs), which were then loaded onto a high-density nanochip. Sequencing was performed using combined probe anchoring and polymerase (cPAS) technology on the high-throughput DNBSEQ™ platform, generating high-quality paired-end sequencing data for subsequent transcriptome analysis.

### 2.4. Processing and Analysis of RNA-Seq Raw Data

Raw sequencing reads were first filtered using SOAPnuke v2.3.2 (https://github.com/BGI-flexlab/SOAPnuke, accessed on 13 January 2026), including the removal of reads shorter than 20 bp, reads containing more than 1% ambiguous bases (“N”), adapter-contaminated reads and reads with more than 50 consecutive identical bases. The resulting high-quality clean reads were used for subsequent analyses. High-quality reads were aligned to the bovine reference genome ARS-UCD 2.0 (https://www.ncbi.nlm.nih.gov/datasets/genome/GCF_002263795.3/, accessed on 24 January 2026) using HISAT2 v2.2.1 (https://daehwankimlab.github.io/hisat2/, accessed on 24 January 2026). Subsequently, clean data was aligned to the reference gene set using Bowtie2 and gene expression quantification was performed using RSEM v1.3.1. Differential expression analysis was performed using DESeq2 in R v4.4.0. Genes with an adjusted *p*-value (padj.) < 0.05 and |log2(fold change)| > 2 were defined as differentially expressed genes (DEGs). Functional enrichment analysis of the identified DEGs was conducted using clusterProfiler v4.6.0 [32] to identify significantly enriched GO terms and KEGG pathways in both upregulated and downregulated gene sets.

### 2.5. Real-Time Quantitative PCR

We selected significantly upregulated and downregulated genes for validation by RT-qPCR. Total RNA was extracted from denuded bovine oocytes at the GV and MII stages using TRIzol reagent (Invitrogen, Carlsbad, CA, USA). Subsequently, RNA was reverse-transcribed into cDNA using HyperScript III RT SuperMix (EnzyArtisan, Shanghai, China) according to the manufacturer’s instructions, and the cDNA was stored at −20 °C until further use. RT-qPCR was carried out using 2 × S6 Universal SYBR qPCR Mix (EnzyArtisan, Shanghai, China) on a real-time PCR system (LongGene, Hangzhou, China). The total reaction volume was 10 μL, comprising 5 μL of 2 × S6 Universal SYBR qPCR Mix, 0.2 μL of forward primer, 0.2 μL of reverse primer, 1 μL of cDNA template, and 3.6 μL of ddH_2_O. The RT-qPCR conditions were as follows: initial denaturation at 95 °C for 30 s, followed by 40 cycles of 95 °C for 10 s, 60 °C for 20 s, and 72 °C for 10 s. Three biological replicates were included for each sample to ensure reliability, and the primers used are listed in Table 1. Based on geNorm analysis, three candidate reference genes (*GAPDH*, *YWHAZ* and *HPRT1*) were evaluated, and the geometric mean of the Cq values of *GAPDH* and *YWHAZ* was used for normalization. Relative mRNA expression levels were calculated using the 2^−ΔΔCt^ method. Statistical analysis was performed using analysis of variance (ANOVA), and graphs were generated using GraphPad Prism 8.0. All results are presented as mean ± standard error of the mean (SEM).

## 3. Results

### 3.1. scRNA-Seq of Bovine Oocytes

**Figure 1:**
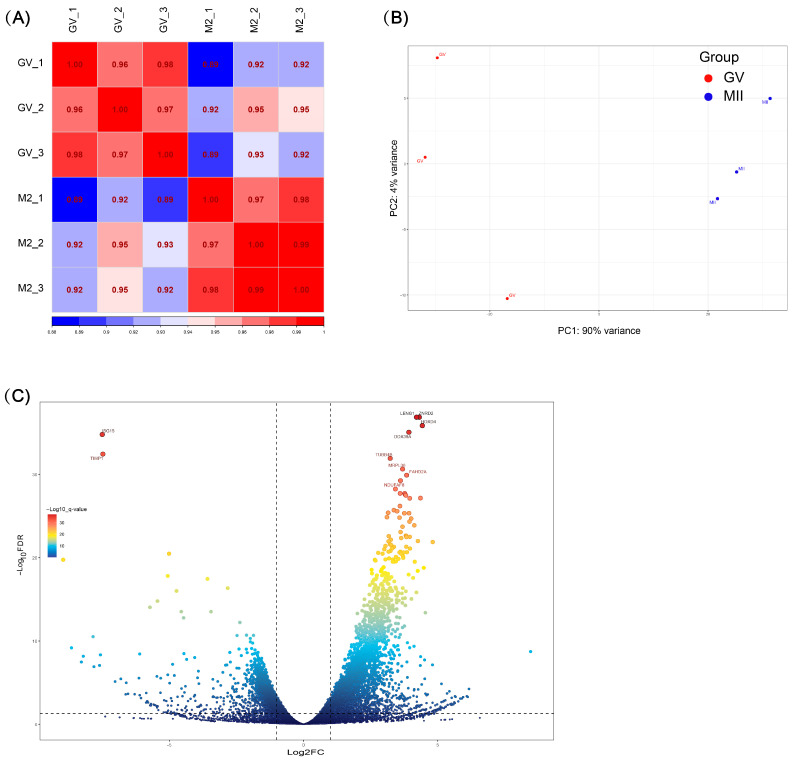
Transcriptomic analysis of bovine oocytes at GV and MII stages. (**A**) Correlation analysis between different samples of bovine oocyte transcriptomes, using Spearman correlation coefficient. (**B**) PCA scatter plot. (**C**) Volcano plot of differentially expressed genes between GV and MII bovine oocytes. Genes on the left, with Padj < 0.05 and log2(fold change) < −2, are significantly downregulated in the GV stage, while genes on the right, with Padj < 0.05 and log2(fold change) > 2, are significantly upregulated in the GV stage. The *X*-axis indicates log2(fold change) and the *Y*-axis indicates −log10(Padj).

After filtering the RNA-seq data, a total of 39.74 GB of clean bases was obtained. High-quality reads were then aligned to the bovine reference genome (ARS-UCD 2.0), and gene-level quantification was performed using RSEM, resulting in a gene count matrix comprising 18,590 annotated genes (Supplementary Table S1). Based on this dataset, inter-sample correlations and principal component analysis (PCA) were performed. The correlation coefficients within GV and MII samples were all greater than 0.96, indicating high consistency of gene expression within each stage. In contrast, the correlation between GV and MII samples ranged from 0.89 to 0.95, suggesting substantial differences in gene expression between these two stages (Figure 1A). PCA revealed a clear separation between GV and MII oocytes along the first principal component (PC1), which explained 90% of the total variance, indicating pronounced transcriptomic differences during oocyte maturation (Figure 1B).

A total of 1787 differentially expressed genes (DEGs) were identified between GV and MII oocytes, including 1556 significantly upregulated and 231 significantly downregulated genes in the GV stage (Figure 1C). Based on the defined thresholds, gene expression patterns differed markedly between the GV and MII stages, indicating extensive transcriptional reprogramming during oocyte maturation (Supplementary Tables S2 and S3).

### 3.2. GO and KEGG Enrichment Analysis of Differentially Expressed Genes

**Figure 2:**
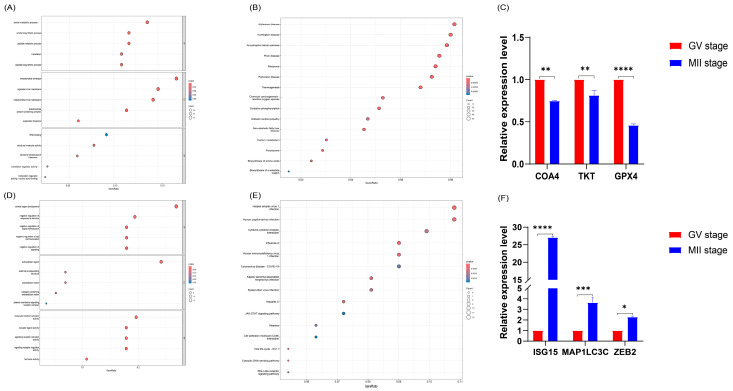
(**A**) GO enrichment analysis of upregulated genes. (**B**) KEGG pathway enrichment analysis of upregulated genes. (**C**) Validation of upregulated genes: *COA4, TKT* and *GPX4*. (**D**) GO enrichment analysis of downregulated genes. (**E**) KEGG pathway enrichment analysis of downregulated genes. (**F**) Validation of downregulated genes: *ISG15*, *MAP1LC3C* and *ZEB2*. In all enrichment plots, the color of the dots represents the statistical significance of enrichment, while the size of the dots indicates the number of genes within each category. “*” indicates *p* < 0.05, “**” indicates *p* < 0.01, “***” indicates *p* < 0.001 and “****” indicates *p* < 0.0001.

To investigate the potential functions of DEGs, functional annotation was performed using the Gene Ontology (GO) and KEGG databases. GO enrichment analysis of upregulated genes revealed significant enrichment in mitochondrial-related terms, including “mitochondrial envelope” and “mitochondrial protein-containing complex” suggesting that mitochondrial energy supply plays a critical role during oocyte maturation (Figure 2A). In addition, numerous genes associated with energy metabolism were significantly enriched among the upregulated gene set. KEGG pathway analysis further demonstrated that upregulated genes were significantly enriched in pathways such as “Oxidative phosphorylation” and “Chemical carcinogenesis—reactive oxygen species” indicating that mitochondrial-associated energy metabolism is essential for oocyte maturation (Figure 2B; Supplementary Table S4).

In contrast, GO enrichment analysis of downregulated genes showed significant enrichment in biological processes such as the negative regulation of signal transduction (Figure 2D). KEGG pathway analysis further revealed that downregulated genes were significantly enriched in pathways including the “JAK-STAT signaling pathway” and “Cell adhesion molecules (CAMs) interaction”. These findings suggest that these pathways may play important roles in maintaining cellular homeostasis and regulating functional transitions during oocyte maturation (Figure 2E; Supplementary Table S5). Overall, these enrichment results highlight substantial transcriptomic differences between GV and MII oocytes and emphasize the critical roles of multiple biological processes and signaling pathways in oocyte maturation.

### 3.3. RT-qPCR Validation of Key Genes

To further validate the expression patterns of key genes associated with oocyte maturation, we performed RT-qPCR on upregulated genes related to mitochondrial function (*COA4*, *TKT* and *GPX4*) and downregulated genes involved in signaling pathways (*ISG15*, *MAP1LC3C* and *ZEB2*). The results showed that *COA4*, *TKT* and *GPX4* exhibited significantly higher expression levels in the GV stage compared to the MII stage (Figure 2C), whereas *ISG15*, *MAP1LC3C* and *ZEB2* displayed significantly lower expression in GV oocytes (Figure 2F). These findings further confirm that the dynamic expression changes in these genes across developmental stages are closely associated with oocyte maturation.

## 4. Discussion

The shift from the GV stage to the MII stage is a pivotal moment in the development of competence, marked not only by meiotic progression but also by coordinated cytoplasmic maturation and the active restructuring of energy metabolism [1]. To explore this, we employed Smart-seq2 to investigate the transcriptomic profiles of bovine oocytes at both the GV and MII stages. Analysis identified 1787 DEGs, revealing distinct expression patterns specific to each developmental stage. Furthermore, functional enrichment analysis showed that these differentially expressed genes were primarily enriched in pathways related to mitochondrial function, energy metabolism, and signal transduction, emphasizing their essential regulatory roles in the maturation of oocytes.

Analysis of functional enrichment indicated that upregulated genes were significantly enriched in pathways associated with mitochondrial activity and energy metabolism, particularly in processes like “oxidative phosphorylation” and “Thermogenesis” (Figure 2B). This finding corresponds with the increased energy demands noted during the maturation of oocytes [33]. Previous studies by Adhikari, D. et al. have shown that pharmacological treatments and mitochondrial supplementation can improve oocyte quality and fertility by enhancing ATP production and reducing ROS levels [34]. The critical role of mitochondria in oocyte maturation has been thoroughly reviewed, highlighting that disruptions in mitochondrial oxidative phosphorylation can lead to decreased ATP production and increased ROS accumulation, ultimately affecting oocyte quality and developmental capacity [35,36]. These findings suggest that the regulation of genes involved in these pathways may represent a key mechanism underlying mitochondrial functional remodeling during oocyte maturation. In our investigation, we validated the expression levels of genes that were upregulated and closely associated with mitochondrial functions; results indicate that *COA4*, *TKT* and *GPX4* are significantly more expressed in GV oocytes compared to MII oocytes (Figure 2C). Cytochrome c oxidase (COX), the terminal complex in the mitochondrial respiratory chain, is responsible for converting oxygen into water while facilitating proton movement to enable oxidative phosphorylation [37,38]. Prior studies have shown that at high ATP/ADP ratios, ATP can inhibit cytochrome c oxidase (CytOx), thereby maintaining mitochondrial membrane potential and significantly reducing ROS production [39]. COA4 is essential in the later stages of Cox1 assembly, particularly in aiding the insertion of copper by Cox11, which is crucial for proper cytochrome c oxidase assembly [40]. Additionally, TKT integrates glucose metabolism with mitochondrial oxidative phosphorylation via the pentose phosphate pathway (PPP), preventing excessive breakdown of fatty acids or amino acids and sustaining mitochondrial balance [41]. A deficiency in TKT results in diminished glycolysis and heightened oxidative stress, leading to excessive catabolism of fatty acids and amino acids, which disrupts oxidative phosphorylation and impairs mitochondrial function [42]. Moreover, antioxidant-related genes such as *GPX4*, *CAT*, *SOD1*, *SOD2* and *SLC25A39* facilitate in vitro oocyte maturation by mitigating ROS-induced lipid peroxidation and enhancing mitochondrial performance [43].

In contrast, GV oocytes exhibited significantly reduced levels of *ISG15*, *MAP1LC3C* and *ZEB2* when compared to MII oocytes, indicating a distinct expression profile that changes with the maturation stages of oocytes. Post-translational modifications (PTMs) play critical roles in oocyte development, with ubiquitination and ubiquitin-like modifications (such as SUMOylation and ISGylation) contributing to meiotic resumption and maintenance of the primordial follicle reserve [44,45]. ISG15, a ubiquitin-like modifier, has been shown to negatively regulate ovulation and female fertility in mammals; its deficiency enhances ovarian responsiveness to gonadotropins and promotes cumulus expansion and ovulation [45]. Additionally, MAP1LC3C is also regulated by post-translational modifications. Previous studies have shown that interactions of LC3 proteins are modulated by PTMs, with phosphorylation at Ser18 affecting the binding affinity of MAP1LC3C to target proteins, thereby influencing autophagic processes [46]. These findings suggest that MAP1LC3C may contribute to the maintenance of cellular homeostasis during oocyte maturation through autophagy-mediated pathways. The transcription factor *ZEB2* regulates cell fate determination and differentiation across multiple cell lineages during embryonic and postnatal development [47].

The findings from this study provide important insights into the molecular mechanisms that govern the maturation of bovine oocytes, particularly in relation to energy metabolism and signaling pathways. However, in this study, the candidate genes were only preliminarily validated by RT-qPCR. Future research should focus on elucidating the specific roles of these candidate genes during oocyte maturation and verifying their functions through gene knockout or overexpression methods.

## 5. Conclusions

In this study, scRNA-seq was employed to comprehensively characterize the transcriptomic profiles of bovine oocytes at the GV and MII stages, identifying a large number of differentially expressed genes associated with oocyte maturation. Further analyses indicated that genes involved in energy metabolism and signal transduction may serve as key regulators of bovine oocyte maturation. Overall, this study provides new insights into the molecular mechanisms underlying bovine oocyte maturation and offers a theoretical foundation for improving oocyte developmental competence and embryonic production efficiency.

## Figures and Tables

**Table 1:** Primer sequence information.

Gene	Forward Primer	Reverse Primer
*COA4*	CCTGGTCCCGACAGGTAAAGA	CACTGCATAGTGGGAAGCAG
*TKT*	GAGCAACATCAACCTCTGCG	TGACGTGGGGATAGACCGAA
*GPX4*	ACCCTCTGTGGAAATGGATG	GAAGGCTTCTCGGAACACAG
*ISG15*	TGACGGTGAAGATGCTAGGG	GCACATTGATCTTCTGGGCG
*MAP1LC3C*	AGTTCCTCAGCGTCATTCGG	TGATCCCAAGCAGCCAAACA
*ZEB2*	ACCCTCCTCTCAGGCTAACA	TGCCAACCTTCGTTTTGCTC
*YWHAZ*	GAGCAGGCTGAGCGATATGA	AAGATGACCTACGGGCTCCT
*GAPDH*	CACCCTCAAGATTGTCAGCA	GGTCATAAGTCCCTCCACGA

## Data Availability

The original contributions presented in the study are included in the article/Supplementary Materials, further inquiries can be directed to the corresponding author.

## Supplementary Materials

The following supporting information can be downloaded at: https://www.mdpi.com/article/10.3390/biology15080662/s1, Supplementary Material Table S1: count matrix; Supplementary Material Table S2: Up-regulated gene set; Supplementary Material Table S3: Down-regulated gene set; Supplementary Material Table S4: Up-regulated genes in GO & KEGG; Supplementary Material Table S5: Down-regulated genes in GO & KEGG.

## References

[1] Pei Z., Deng K., Xu C., Zhang S. (2023). The molecular regulatory mechanisms of meiotic arrest and resumption in Oocyte development and maturation. Reprod. Biol. Endocrinol..

[2] Caniçais C., Vasconcelos S., Santos F., Dória S., Marques C.J. (2025). DNA methylation mechanisms in the maturing and ageing oocyte. Epigenetics Chromatin.

[3] Duan J., Xu A., Xie J., Wang L., Lv Q., Yang J., Zhao N., Li B., Cao G. (2025). GPR30-mediated the MEK/ERK signaling pathway promotes porcine oocyte maturation and development. Theriogenology.

[4] Huelgas-Morales G., Greenstein D. (2018). Control of oocyte meiotic maturation in *C. elegans*. Semin. Cell Dev. Biol..

[5] Zhu S., Wang Q. (2022). Metabolic control of oocyte development. Biol. Reprod..

[6] Pietroforte S., Martins M., Barragan M., Ibañez E., Vassena R., Popovic M., Sanchez T., Sakkas D., Zambelli F. (2025). Mitochondrial metabolism influences meiotic maturation in human oocytes of young and advanced maternal age women. Hum. Reprod..

[7] Russell D.L., Gilchrist R.B., Brown H.M., Thompson J.G. (2016). Bidirectional communication between cumulus cells and the oocyte: Old hands and new players?. Theriogenology.

[8] Del Bianco D., Gentile R., Sallicandro L., Biagini A., Quellari P.T., Gliozheni E., Sabbatini P., Ragonese F., Malvasi A., D’Amato A. (2024). Electro-Metabolic Coupling of Cumulus-Oocyte Complex. Int. J. Mol. Sci..

[9] Jiang Y., Adhikari D., Li C., Zhou X. (2023). Spatiotemporal regulation of maternal mRNAs during vertebrate oocyte meiotic maturation. Biol. Rev. Camb. Philos. Soc..

[10] Qin J., Wei Y., Ning A., Hu W., Wan P., Cao B., Pan B., Lv T., Du K., Yao X. (2025). Decoding the Molecular Landscape of Prepubertal Oocyte Maturation: GTPBP4 as a Key Driver of In Vitro Developmental Competence. Cell Prolif..

[11] Hu W., Zeng H., Shi Y., Zhou C., Huang J., Jia L., Xu S., Feng X., Zeng Y., Xiong T. (2022). Single-cell transcriptome and translatome dual-omics reveals potential mechanisms of human oocyte maturation. Nat. Commun..

[12] Sha Q.Q., Yu J.L., Guo J.X., Dai X.X., Jiang J.C., Zhang Y.L., Yu C., Ji S.Y., Jiang Y., Zhang S.Y. (2018). CNOT6L couples the selective degradation of maternal transcripts to meiotic cell cycle progression in mouse oocyte. EMBO J..

[13] Takeuchi H., Yamamoto M., Fukui M., Inoue A., Maezawa T., Nishioka M., Kondo E., Ikeda T., Matsumoto K., Miyamoto K. (2022). Single-cell profiling of transcriptomic changes during In Vitro maturation of human oocytes. Reprod. Med. Biol..

[14] Yu B., Doni Jayavelu N., Battle S.L., Mar J.C., Schimmel T., Cohen J., Hawkins R.D. (2020). Single-cell analysis of transcriptome and DNA methylome in human oocyte maturation. PLoS ONE.

[15] Xiong X.R., Lan D.L., Li J., Zi X.D., Li M.Y. (2016). Identification of candidate miRNAs and expression profile of yak oocytes before and after In Vitro maturation by high-throughput sequencing. Reprod. Domest. Anim..

[16] Vallée M., Aiba K., Piao Y., Palin M.F., Ko M.S., Sirard M.A. (2008). Comparative analysis of oocyte transcript profiles reveals a high degree of conservation among species. Reproduction.

[17] Biase F.H. (2017). Oocyte Developmental Competence: Insights from Cross-Species Differential Gene Expression and Human Oocyte-Specific Functional Gene Networks. OMICS J. Integr. Biol..

[18] Richani D., Dunning K.R., Thompson J.G., Gilchrist R.B. (2021). Metabolic co-dependence of the oocyte and cumulus cells: Essential role in determining oocyte developmental competence. Hum. Reprod. Update.

[19] Imanaka S., Shigetomi H., Kobayashi H. (2022). Reprogramming of glucose metabolism of cumulus cells and oocytes and its therapeutic significance. Reprod. Sci..

[20] Eppig J.J. (1976). Analysis of mouse oogenesis In Vitro. Oocyte isolation and the utilization of exogenous energy sources by growing oocytes. J. Exp. Zool..

[21] Pandey A.N., Yadav P.K., Premkumar K.V., Tiwari M., Pandey A.K., Chaube S.K. (2024). Reactive oxygen species signalling in the deterioration of quality of mammalian oocytes cultured In Vitro: Protective effect of antioxidants. Cell Signal..

[22] Kala M., Shaikh M.V., Nivsarkar M. (2017). Equilibrium between anti-oxidants and reactive oxygen species: A requisite for oocyte development and maturation. Reprod. Med. Biol..

[23] Macaulay I.C., Voet T. (2014). Single cell genomics: Advances and future perspectives. PLoS Genet..

[24] Caniçais C., Sobral D., Vasconcelos S., Cunha M., Pinto A., Mesquita Guimarães J., Santos F., Barros A., Dória S., Marques C.J. (2025). Transcriptomic analysis and epigenetic regulators in human oocytes at different stages of oocyte meiotic maturation. Dev. Biol..

[25] Jiao Y., Gao B., Wang G., Li H., Ahmed J.Z., Zhang D., Ye S., Liu S., Li M., Shi D. (2020). The key long non-coding RNA screening and validation between germinal vesicle and metaphase II of porcine oocyte In Vitro maturation. Reprod. Domest. Anim..

[26] Wang J.J., Niu M.H., Zhang T., Shen W., Cao H.G. (2020). Genome-Wide Network of lncRNA-mRNA During Ovine Oocyte Development From Germinal Vesicle to Metaphase II In Vitro. Front. Physiol..

[27] Li Z., Song X., Yin S., Yan J., Lv P., Shan H., Cui K., Liu H., Liu Q. (2021). Single-Cell RNA-Seq Revealed the Gene Expression Pattern during the In Vitro Maturation of Donkey Oocytes. Genes.

[28] Wang N., Li C.Y., Zhu H.B., Hao H.S., Wang H.Y., Yan C.L., Zhao S.J., Du W.H., Wang D., Liu Y. (2017). Effect of vitrification on the mRNA transcriptome of bovine oocytes. Reprod. Domest. Anim..

[29] Huang J., Ma Y., Wei S., Pan B., Qi Y., Hou Y., Meng Q., Zhou G., Han H. (2018). Dynamic changes in the global transcriptome of bovine germinal vesicle oocytes after vitrification followed by In Vitro maturation. Reprod. Fertil. Dev..

[30] Hao T., Xu X., Hao H., Du W., Pang Y., Zhao S., Zou H., Yang S., Zhu H., Yang Y. (2021). Melatonin improves the maturation and developmental ability of bovine oocytes by up-regulating GJA4 to enhance gap junction intercellular communication. Reprod. Fertil. Dev..

[31] Diaz F.A., Gutierrez-Castillo E.J., Foster B.A., Hardin P.T., Bondioli K.R., Jiang Z. (2021). Evaluation of Seasonal Heat Stress on Transcriptomic Profiles and Global DNA Methylation of Bovine Oocytes. Front. Genet..

[32] Yu G., Wang L.G., Han Y., He Q.Y. (2012). clusterProfiler: An R package for comparing biological themes among gene clusters. OMICS J. Integr. Biol..

[33] Placidi M., Di Emidio G., Virmani A., D’Alfonso A., Artini P.G., D’Alessandro A.M., Tatone C. (2022). Carnitines as Mitochondrial Modulators of Oocyte and Embryo Bioenergetics. Antioxidants.

[34] Adhikari D., Lee I.W., Yuen W.S., Carroll J. (2022). Oocyte mitochondria-key regulators of oocyte function and potential therapeutic targets for improving fertility. Biol. Reprod..

[35] Kirillova A., Smitz J.E.J., Sukhikh G.T., Mazunin I. (2021). The Role of Mitochondria in Oocyte Maturation. Cells.

[36] van der Reest J., Nardini Cecchino G., Haigis M.C., Kordowitzki P. (2021). Mitochondria: Their relevance during oocyte ageing. Ageing Res. Rev..

[37] Gladyck S., Aras S., Hüttemann M., Grossman L.I. (2021). Regulation of COX Assembly and Function by Twin CX_9_C Proteins—Implications for Human Disease. Cells.

[38] Čunátová K., Reguera D.P., Houštěk J., Mráček T., Pecina P. (2020). Role of cytochrome c oxidase nuclear-encoded subunits in health and disease. Physiol. Res..

[39] Ramzan R., Dolga A.M., Michels S., Weber P., Culmsee C., Rastan A.J., Vogt S. (2022). Cytochrome c Oxidase Inhibition by ATP Decreases Mitochondrial ROS Production. Cells.

[40] Swaminathan A.B., Soma S., Vicary A.C., Zulkifli M., Kaur H., Gohil V.M. (2022). A yeast suppressor screen links Coa4 to the mitochondrial copper delivery pathway for cytochrome c oxidase. Genetics.

[41] Chen Z., Wang Y., Tong X. (2025). Transketolase in metabolic diseases, autoimmune diseases and cancer. Methods Enzymol..

[42] Liu Q., Zhu F., Liu X., Lu Y., Yao K., Tian N., Tong L., Figge D.A., Wang X., Han Y. (2022). Non-oxidative pentose phosphate pathway controls regulatory T cell function by integrating metabolism and epigenetics. Nat. Metab..

[43] Gao L., Zhang C., Zheng Y., Wu D., Chen X., Lan H., Zheng X., Wu H., Li S. (2023). Glycine regulates lipid peroxidation promoting porcine oocyte maturation and early embryonic development. J. Anim. Sci..

[44] Li H., You L., Tian Y., Guo J., Fang X., Zhou C., Shi L., Su Y.Q. (2020). DPAGT1-Mediated Protein N-Glycosylation Is Indispensable for Oocyte and Follicle Development in Mice. Adv. Sci..

[45] Chen Y., Zhou J., Wu S., Wang L., Chen G., Chen D., Peng X., Miao Y.L., Mei S., Li F. (2023). ISG15 suppresses ovulation and female fertility by ISGylating ADAMTS1. Cell Biosci..

[46] Krichel C., Möckel C., Schillinger O., Huesgen P.F., Sticht H., Strodel B., Weiergräber O.H., Willbold D., Neudecker P. (2019). Solution structure of the autophagy-related protein LC3C reveals a polyproline II motif on a mobile tether with phosphorylation site. Sci. Rep..

[47] Birkhoff J.C., Korporaal A.L., Brouwer R.W.W., Nowosad K., Milazzo C., Mouratidou L., van den Hout M.C.G.N., van IJcken W.F.J., Huylebroeck D., Conidi A. (2023). Zeb2 DNA-Binding Sites in Neuroprogenitor Cells Reveal Autoregulation and Affirm Neurodevelopmental Defects, Including in Mowat-Wilson Syndrome. Genes.

